# Efficacy of Parenting Education Compared to the Standard Method in Improvement of Reading and Writing Disabilities in Children

**Published:** 2014

**Authors:** Mojgan Karahmadi, Fereshteh Shakibayee, Hushang Amirian, Reza Bagherian-Sararoudi, Mohammad Reza Maracy

**Affiliations:** 1 Associate Professor, Department of Psychiatry, School of Medicine, Isfahan University of Medical Sciences, Isfahan, Iran.; 2 Associate Professor, Department of Psychiatry, School of Medicine, Kermanshah University of Medical Sciences, Kermanshah, Iran.; 3 Associate Professor, Department of Epidemiology and Biostatics, School of Health, Isfahan University of Medical Sciences, Isfahan, Iran.

**Keywords:** Learning Disability, Parenting Education, Reading Disability, Writing Disability

## Abstract

**Objective:** The present study aimed to evaluate the effect of parenting education on improvement of reading and writing disabilities in children.

**Methods:** A randomized controlled trial was done on primary school students with reading and writing disabilities and their mothers. The subjects were divided into three groups with 26 members in each group. The first group (mothers’ education group) received 6 one-hour new educational sessions. The second group (standard group) received 12-15 standard educational sessions for learning disability, and the third group (control group) which consisted of students with learning disability did not receive any treatments. Research instruments included reading and writing tests, and demographic questionnaire. The three groups were evaluated via pretest and posttests at baseline and after one and three months of educational interventions. Data were analyzed using the chi-square, t-test, and repeated measures multivariate analysis of variance (MANOVA).

**Results: **The mean reading speed had the most progression in the mothers' education group. Comparison among reading speed, reading accuracy, and spelling scores has been statistically significant (F 2, 6 = 90.64;p < 0.001) but the mean of these scores has been insignificant among the three groups (F 2, 67 = 0.583;p > 0.05). The mean reading accuracy, mostly increased after 3-month interventions in the mothers group. The control group had the lowest mean reading accuracy scores.

**Conclusion:** Parenting education in mothers had a positive effect on the treatment of children with reading and writing disabilities.

**Declaration of interest:** None.

**Clinical Trial Registration-URL: **http://www.irct.ir. Unique identifier: IRCT201101205653N1.

## Introduction

Communication is one of the most basic aspects of development. Communication is not limited to speech and includes any kind of sending messages to others such as writing. Writing is the most developed and complex aspect of communication (1). Writing and reading are communication skills which are learnt in the first years of primary school and developed in the following years. Writing and reading are indispensable (1).

According to the Diagnostic and Statistical Manual of Mental Disorders, 4^th^ edition (DSM-IV) criteria, learning disability is defined as decrease in the rate of success in standardized reading and writing tests which is significantly lower than the normal results based on age, education, and intelligence quotient (IQ) (1-3).

About 6-12% of the students suffer from learning disability with higher prevalence in boys (3). Genetic and metabolic disorders, poverty, malnutrition, lead poisoning, low birth weight, and social exclusion are the possible causes of learning disability (1, 3-5).

The most common types of learning disorder are reading, writing, and mathematics disorders (3, 6). Learning disabilities interfere with school performances such as listening, thinking, speaking, reading, spelling, and writing (1, 3). Higher rate of school drop outstand job difficulties despite normal IQ, are due to delay in diagnosis and treatment of learning disability (1). Despite normal IQ, these children cannot understand the words. This condition is known as latent retardation and may lead to despair and lack of self-esteem (2).

Children with learning disability have moderate to above moderate IQ in several functional fields, but have special defects in narrow fields of academic skills (3).

Common and useful treatment modalities for learning disability include individualized educational programs, multi-sensational method (Fernald), experiences repetition, and response treatment intervention (RTI) (3, 7-9).

The results of several studies have shown that providing appropriate information about learning disability to parents can improve parents-child relationship. Parents will understand that these children do not have low IQ or organic defect, so should not feel despair or blame themselves (4).

Studies have reported that 15% of primary-school students with reading disability in Iran have mild sensation and movement disorders (10). Specialists recommend parents must be educated about the nature of learning disability and as many as available special education methods (5, 11). Educational support of the family is one of the key factors in the treatment of learning disabilities (3, 5, 11). Parent’s management training has been recommended in treatment of reading and writing disabilities in children. In this method, a behavioral management program for child improves the educational programs (12). Results of a study showed that children with learning disabilities had better function after receiving their parents’ education and supervision (13).

According to the importance of parent’s education in the improvement of learning disability and as the reading and writing disabilities are the most common types of learning disability, this study aimed to evaluate the effect of parenting education compared to the standard method in treatment of reading and writing disability in their children.

## Materials and Methods

This was a randomized controlled trial study on 78 second grade primary school students with diagnosis of reading and writing disability according to the DSM-VI criteria (1) who were referred from their schools with their mothers as consecutive cases to two learning consultation centers of the Education Department in Isfahan, Iran.

At first, the students were interviewed by a child and adolescent psychiatrist, and then they were assessed through the Wechsler Child Intelligence Scale (WISC) by psychologists of the studied centers. Students with normal IQ were evaluated via the Reading and Writing Test (11) and the study subjects were selected according to the inclusion and exclusive criteria. The IQ test was performed according to the DSM-IV criteria for diagnosis of learning disability which includes students with normal IQ (1).Wechsler Intelligence Test was used for the assessment of IQ. The inclusive criteria for the students were diagnosis of reading and writing disability according to the DSM-IV criteria, no family problems (divorce, separation, or adoption), and also no deafness, blindness, paralysis, or developmental disorder. Students with any mental retardation or borderline IQ were excluded.

Inclusive criteria for the mothers were being at high school graduates. The written consent was obtained from all mothers whose children were enrolled in this study. The Reading and Writing Test had reliability of about 95% and a validity of about 87% and was confirmed by the Pediatric Psychiatry and Rehabilitation professors of Tehran and Isfahan Universities of Medical Sciences (14). The Reading test evaluated the speed and accuracy of reading while the spelling test evaluates the number of correct written words (14). The reading test has been normalized on the Iranian students and includes nine variables which contain nine common mistakes in reading disability. The scores of the mean, standard deviation (SD), and percentiles of the accuracy and speed of reading, have been made separately for each of the selected texts. Using the above mentioned criteria, child’s reading status could be evaluated (14). In reading test, accuracy and speed of reading were calculated via the following formulas:

*Reading speed = 51⁄reading time × 60*

*Reading accuracy = sum of mistakes*

The spelling test has been normalized for the Iranian students and the content has been arranged from simple words to more complex words. The correct words were obtained from the test and then the score (%) will be calculated via the following formula (14). 

*Score = (number of correct words ⁄25) × 100*

In this study, the assessment check lists including reading score check list (both speed and accuracy of reading) and spelling scores check lists were used. The subjects were randomly divided into three groups using random numbers table: “mothers (intervention) group”, “standard group”, and “control group”. In intervention group, mothers of the students formed a group with 26 members and received 6 one-hour educational sessions according to the educational protocol including parenting method, reading and writing special education methods. Then these mothers educated their children for 3 months. Mothers’ education sessions were as follows:

The first session i.e. the introduction to reading and writing disability (by child and adolescent psychiatrist).

The second session i.e. how can parents help child with learning disability? (by child and adolescent psychiatrist).

The third session i.e. positive communication with child (by child and adolescent psychiatrist).

The fourth session i.e. education of reward and stress control methods (by child and adolescent psychiatrist).

The fifth session i.e. special education of reading, development of visual and hearing memory (by specialist of learning disability education).

The sixth session i.e. special education of writing (by specialist of learning disability education).

After the sixth session, the mothers took a post-test about their degree of satisfaction from educational sessions. Then mothers taught their children in 12 to 15 educational sessions and their children were evaluated at baseline, 1, and 3 months after the educational interventions. The standard group included 26 students with reading and writing disability who were educated individually (routine method of learning centers) in 12 to 15 educational sessions. These children were evaluated at baseline, 1, and 3 months after the educational interventions. The control group included 26 students with reading and writing disability in waiting lists to attend educational centers. No treatment modalities to address the disabilities were initiated, but later they were put prior to other patients in waiting lists of learning centers. The students whose mothers were absent from at least two educational sessions were excluded.

Data were analyzed using the SPSS for Windows 11.5 (SPSS Inc., Chicago, IL, USA). The chi-square test was used for the comparison of qualitative demographic variables among groups and the t-test was used to compare quantitative demographic variables. In this study, the required defaults included normal distribution and equal variance for spelling scores, reading accuracy and reading speed at baseline, 1, and 3 months after intervention in three studied groups.

The Levene’s test was used for control of the efficacy of variables. The repeated measures multivariate analysis of variance (MANOVA) was used for comparison among the three groups.

## Results

The selected students comprised of 51 boys (65%) and 27 girls (35%) girls. Mean age of the students was 7.4 years in the intervention group, 7.5 years in the standard group, and 7.4 years in the control group. Mean age of the mothers in this study was 37.3 years and all of them were high school graduates. Two students from the standard group and two members from the control group were excluded because of immigration and familial problems. Three mothers were excluded because of absences from more than two educational sessions.

Table 1 illustrates that the mean reading speed score (numbers of read words in one minute) had a 44.2% progression in the mothers group and 22.7% in the standard group. The reading speed compared with the mean spelling score and reading accuracy had the most progression in the mothers group. The reading accuracy scores had 24% progression in the mothers group and 20% in the standard group, but only about 8% progression in the control group which was 1/3 of progression in the mothers group (Table 1).

The results showed that the mean spelling scores has increased about 25% in the mothers group, 15% in the standard group, and 10.4% in the control group, after 3 months. The mean baseline spelling scores before the educational sessions was 8% lower than in the mothers group compared to the two other groups (Table 1).

Table 2 shows that comparison among reading speed, reading accuracy and spelling scores of pretest and posttests have been statistically significant (F 2,66 = 90.64;p < 0.001), but the mean of these scores has been statistically insignificant among the three studied groups (F 2,67 = 0.583; p > 0.05).

**Figure1:**
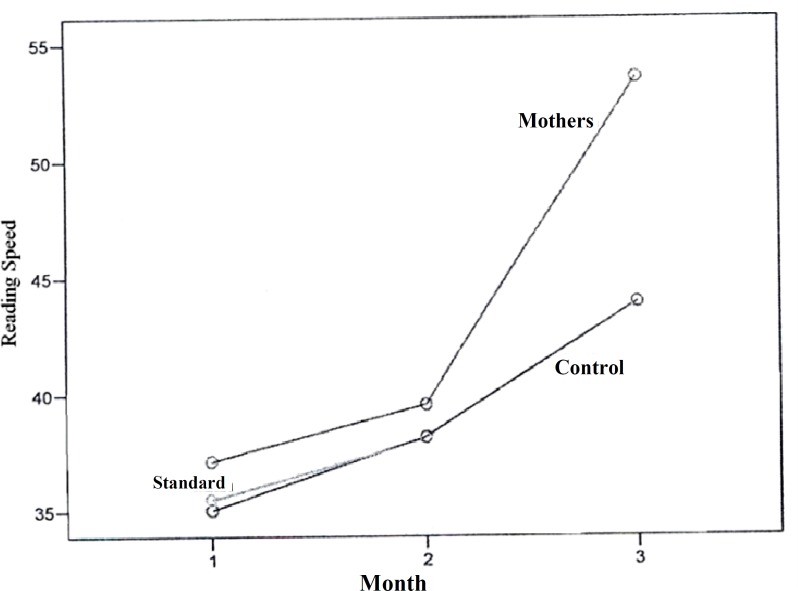
Comparison of reading speed among the three studied groups at baseline, one month and three months after the educational interventions

**Figure 2:**
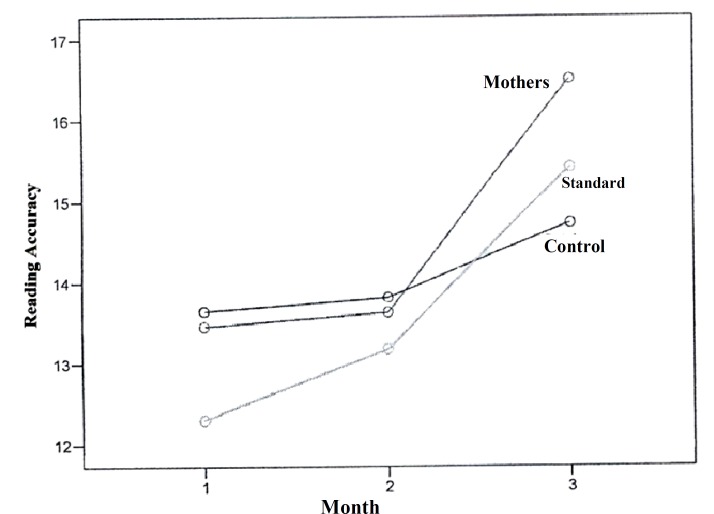
Comparison of reading accuracy among three studied groups at baseline, one month and three months after the educational interventions

**Figure 3:**
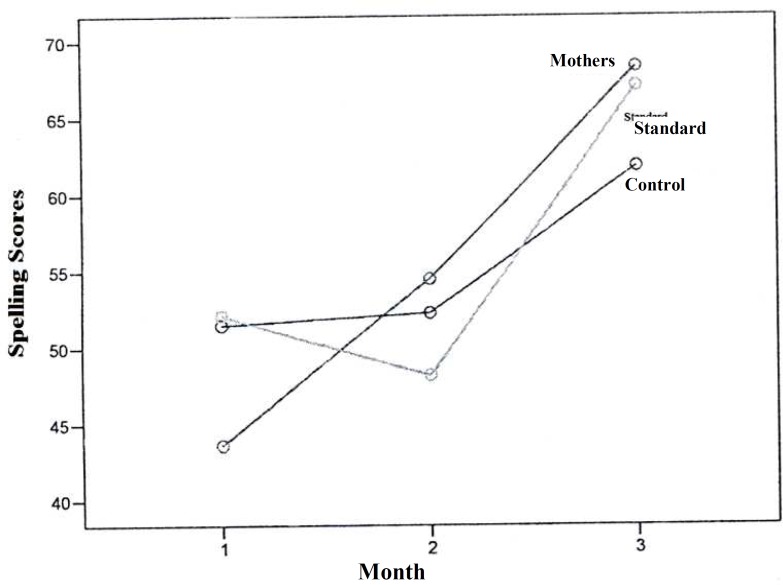
Comparison of spelling scores among the three studied groups at baseline, one month and three months after the educational interventions

In this study, the mean reading speed did not show any statistical significant changes in the studied groups one month after the interventions. However, it increased significantly in the mothers group compared to the two other groups, three months after the interventions, and this significant difference increased during this time (Figure 1). The mean reading accuracy mostly increased after 3 months of interventions in the mothers group compared to the two other groups and this difference increased during this time. The control group had the lowest mean of reading accuracy compared with the two other groups and this difference increased during this time (Figure 2). Mean spelling scores in the mothers’ education group at the baseline was lower compared to the two other groups and had a steady progression during this time. In the standard group, the mean spelling scores decreased after one month and then showed a progressive course. Mean spelling scores in the control group had no change after one month, but had a slower progression during this time in comparison with the two other groups (Figure 3).

**Table 1:** Frequency, mean and standard deviation (SD) of reading speed, reading accuracy and spelling errors scores in mothers’ education, standard method and control groups

**Groups**			**Mean**	**Standard deviation**	**Number**
**Reading** **Speed scores(the number of words in a minute)**	**Baseline**	**Control**	35.0	35.0	24
		**Mothers**	37.1	37.1	23
		**Standard**	35.4	34.4	23
		**Total**	35.8	35.8	70
	**1 month later**	**Control**	38.1	38.1	24
		**Mothers**	38.0	36.5	23
		**Standard**	38.5	38.0	23
		**Total**	38.6	35.5	70
	**3 months later**	**Control**	43.9	43.9	24
		**Mothers**	53.5	53.5	23
		**Standard**	43.8	43.8	23
		**Total**	47.6	47.6	70
					
**Reading accuracy scores**	**Baseline**	**Control**	13.6	13.6	24
		**Mothers**	13.4	13.4	123
		**Standard**	12.3	12.3	23
		**Total**	13.1	13.1	70
	**1 month later**	**Control**	13.8	13.8	24
		**Mothers**	13.6	13.6	23
		**Standard**	13.1	13.1	23
		**Total**	13.5	13.5	70
	**3 months later**	**Control**	14.7	14.7	24
		**Mothers**	16.5	16.5	23
		**Standard**	15.4	15.4	23
		**Total**	15.5	15.5	70
					
**Percent of spelling scores**	**Baseline**	**Control**	51.4	11.4	24
		**Mothers**	43.4	12.9	23
		**Standard**	52.0	13.2	23
		**Total**	49.0	12.9	70
	**1 month later**	**Control**	52.2	12.4	24
		**Mothers**	54.4	11.9	23
		**Standard**	48.4	12.2	23
		**Total**	51.4	12.3	70
	**3 months later**	**Control**	61.8	15.2	24
		**Mothers**	68.3	16.2	23
		**Standard**	67.0	13.3	23
		**Total**	65.0	14.9	70

**Table 2:** Comparison among reading speed, reading accuracy and spelling scores in three time stages(baseline, one month later and three months later) in three studied groups using MANOVA-repeated measures

**Fallow ups and groups**	**Wilks’ lambda**	**F**	**df**	**P-value**
**Fallow ups at baseline, one month and three months later**	0.26	90.64	2.66	< 0.001
**Groups: control, mothers education, and standard**	-	0.58	2.67	0.560

P-value < 0.05 is considered significant

In our study, mean reading speed, reading accuracy, and spelling error scores were higher in mothers who have been trained about the learning disability compared to the standard and the control groups. The reading speed had a two-fold improvement and spelling progressed about 1.5 times in the mothers group compared with the two other groups in this study. The mean increase in reading accuracy scores was similar in the mothers and the standard group and about three-fold of the control group. Mean of the pre-test scores, and post-test scores, one-month and three-months after the educational sessions, significantly increased (p < 0.001) and this increase was more significant over time.

## Discussion

This study aimed to compare the efficacy of parenting education for mothers with standard method in improvement of reading and writing disability in their children. According to 6-12% prevalence of learning disabilities which has negative effect on educational progression and social activities, paying attention to the treatment of learning disabilities is very important (3). Fallah Chay (15) on the epidemiology of reading and writing disability in primary school students in Iran showed that the prevalence of reading and writing disorder is about 11.2% which is close to the results of other studies in other parts of the world which is between 6-12% (3). 

Special education approach is the first line treatment in children with learning disability and other treatment modalities like medications are considered as complementary methods (3).

Kenny and McGilloway showed that parents who have a child with learning disability experienced more stress and less support and required appropriate education about learning disability (11). Studies have shown that parents who have a child with learning disability need at least 4-6 hours educational sessions per day about the nature of learning disability and special education methods (5, 11). Arman and Ghafghazi have suggested that management education for parents can decreases the severity of attention deficit hyperactivity disorder (ADHD) and conduct disorder in their children (16). Motamedi et al. showed that parents’ education is an effective behavioral adjustment method in the disruptive behavior disorder in children (17). Zahed concluded that couples conversation techniques is an acceptable method for problem solving in couples in Iran and has a positive effect on children's behavior (18).

Although these studies were not about learning disability; however, all of them showed positive effect of parents’ education on children behavior which are close to our own study results which showed mothers education has positive effect on the treatment of learning disability. Our study had limitations such as small sample size and short duration of the study.

## Conclusion

This study concluded that parenting education in mothers who had children with reading and writing disabilities had a positive effect on the improvement of the reading and writing disabilities compared to children whose mothers did not receive any proper education about learning disability. Further studies on larger sample sizes and longer period of time (more than 3 months) are suggested for the better evaluation of the effect of parenting education on reading and writing disabilities.
